# Sulphuric Acid Treatment of Pulpectomy

**Published:** 1910-06-15

**Authors:** R. Kern


### SULPHURIC ACID TREATMENT OF PULPECTOMY.

BY R. KERN.

(*L'Art Dentaire*, Bordeaux, France, Nov. 1909.)

The good results which I have obtained in caries of the fourth degree with sulphuric acid treatment have induced me to present you with this article.

This therapeutic treatment is very simple for single-rooted teeth and for upper bicuspids; but, it offers some difficulties for molars especially those which are affected with distal cavities.

*In cases of single-rooted teeth and bicuspids.*—The precautions indicated by Dr. Siffre are to be carefully followed, *i. e.*, the mucous membrane should be protected from the drug. For this process, a copper sound is used, upon the end of which cotton has been twisted and dipped into the acid. It is then brought in contact with the pulp and with gentle pressure, it is forced into the root-canal; but this must take place very slowly and very gradually. Very little pain is usually caused by this treatment, and it generally takes place when the acid comes in contact with the pulp. If there is intense pain experienced when the sound penetrates the root-canal it is usually caused by a too small quantity of sulphuric acid, and in such cases, the sound should be, with the cotton, dipped in the acid and the operation continued. When the sound has reached the apex of the root, it should be rotated and withdrawn. This in most cases will remove all contents of root-canal without any manifestation of pain from the patient.

*In cases of molars.*—I proceed in the following manner, after the mucous membrane surrounding the tooth has been protected. The cavity should be cleansed and the pulp denuded or exposed, then a pledget of cotton saturated with sulphuric acid is placed on the pulp and allowed to remain there for a few seconds and sometimes one minute, according to the case. This will enable the operator to remove the pulp from the pulp-chamber, after which each root may be treated as above mentioned as if it were a single rooted-tooth.—*American Journal*, March.

